# Eating Behaviour and Physical Fitness in 10-Year-Old Children Attending General Education and Sports Classes

**DOI:** 10.3390/ijerph17186467

**Published:** 2020-09-05

**Authors:** Katarzyna Ługowska, Wojciech Kolanowski, Joanna Trafialek

**Affiliations:** 1 Faculty of Medical and Health Sciences, Siedlce University, 08-110 Siedlce, Poland; katarzyna.lugowska.zdoz@uph.edu.pl; 2 Institute of Human Nutrition Sciences, Warsaw University of Life Sciences, 02-787 Warsaw, Poland; joanna_trafialek@sggw.pl

**Keywords:** BMI, children health, Eurofit, fitness, sports classes

## Abstract

The aim of this study was to evaluate the body mass index (BMI), selected eating behaviour and physical fitness of children aged 10 years attending general education and sports classes in Siedlce. Subject children were 272 girls and boys mean aged 10.8-years attending general education (GC) and sports classes (SC). Survey questionnaires consisted of 18 questions about eating behaviour and physical activity. The BMI was determined for each child and compared with reference percentile charts. Eurofit testing was used to measure physical fitness. Increased physical fitness was positively correlated with beneficial eating behaviour among children. SC children showed significantly more frequent dietary intakes of milk, dairy products, poultry, fish, wholegrain bread, groats and vegetables when compared to GC ones. However, significantly more GC children ate red meat more frequently along with snacking on confectionery and savouries than SC ones. Most subjects fell within correct BMI percentile ranges. Underweight was more frequent in SC children at 12.85% than in GC children 9.88%. Overweight and obesity was most often observed in GC children (respectively 19.73% and 5.51%) compared to SC ones (respectively 14.37% and 3.8%). SC children achieved significantly better results in the Eurofit tests. The highest levels of physical fitness and most favourable BMIs and eating behaviour were observed more often among SC children than GC ones. The results confirmed the beneficial health effects of physical fitness for children.

## 1. Introduction

Sport is one of the most beneficial forms of physical activity for children [1]. Many studies have shown physical fitness to be a key indicator of health in both childhood and adulthood [2,3]. Optimal fitness levels in school children are considered as an important factor in preventing lifestyle-related diseases. The World Health Organization (WHO) promotes a healthy lifestyle which includes regularly sport activities [4]. It also recommends that regular monitoring of fitness should be considered as a public health priority. Furthermore, organised forms of physical activity should make up an indispensable part of a child’s daily activity. According to current recommendations, children aged 5–17 should undertake medium or high intensity physical activity lasting at least 60 min each day, while limiting the time spent on using television, computers and telephones to two hours a day [4,5]. Physical activity lasting over 60 min will provide additional health benefits. Intensive activities, including muscle and bone strengthening, should be performed at least three times a week. It is worth noting that for school children, activities related to loading bones can be performed as part of games, running or jumping. It was shown that children following such recommendations stand to gain health benefits [6]. Higher levels of cardio-respiratory performance, along with muscle strength and body composition in childhood and adolescence, are associated with a healthier cardiovascular profile and lower risk of death later in life. Therefore, adhering to and controlling balanced eating habits along with good physical activity levels are important factors for current and future health [7]. 

In addition to physical activity, the diet is another important factor affecting health. The Western style diet (i.e., diet rich in saturated fat and red meat and poor in fresh fruit and vegetables) has been correlated with many negative health consequences (i.e., obesity, hypertension, heart disease and hypercholesterolemia), so the Mediterranean diet (MD) is considered for one of the elements of health [8,9]. The MD concept is based on the use of olive oil, eating of vegetables, fruit, whole grains, nuts and seeds, and moderate eating of legumes. Fish and white meat have priority over processed and red meat. This diet is associated with a balanced and varied diet, providing the majority of the necessary macronutrients in the right proportion [10]. Studies show a positive relationship between healthy diet and sports activity [11,12,13,14,15,16]. 

The WHO states that engaging in organised sport during childhood and adolescence plays an important role for reducing the global epidemic of obesity. They emphasise the important role of sports for encouraging youngsters to change their behaviour, so as to take care of their own health [17,18,19,20,21]. In addition, those children who are heavily engaged in sport are more likely to continue doing so in their adulthood [22]. 

Despite sport being popular amongst children, there is evidence showing that interest in sports often declines, especially during puberty [20,22,23]. This being observed more frequently in girls than boys [24,25,26] estimated that worldwide, ca. 80% of 13–15 year-old teenagers do not comply with WHO guidelines on meeting targets of required physical activity. The study by [27] showed that ‘average grades’ of physical activity in children and adolescents are globally low [27]. 

It should be stressed that children customarily experience their first contacts with systematic forms of physical activity at primary school; especially those attending the so-called ‘sports classes’, which allot a greater amount of time devoted to physical activity and games that are result-achievement orientated than general classes. Moreover, appropriate nutrition, over and above the requirements for growth and development, is equally significant for achieving success in sports [28,29]. 

Standardised methods are used to evaluate physical fitness in schoolchildren. Many measures and tests are used for determining physical fitness in children and adolescents, as well as in contestants engaged in various sporting disciplines; there being over 15 groups of tests currently available for assessing physical fitness [30]. At present, Eurofit, Fitnessgram, Indares, Ovov i Unifittest are the ones most commonly used. 

The European Council’s Committee for the Development of Sport created a European Test of Physical Fitness (Eurofit) to allow outcomes to be compared between different countries through using standard methodologies [31,32]. The test is divided into two parts: one part for children and another part for adolescents [33,34]. Tested dimensions of physical fitness include agility, strength, endurance of the musculature and the cardiovascular system, flexibility, speed and balance. Such tests can be conveniently organised and carried out at schools. 

It was shown that children and adolescents aged 9–17 years have demonstrated exceptionally low levels of physical fitness, particularly in South American and Southern European countries [35]. The study by [36] showed that more children in Greece fell within the lowest aerobic quartile in girls (from 21% to 48.2%) and from 25.7% to 38.7% in boys during an 11 year period (1997–2007). Promoting physical activity in childhood is therefore the key importance to public health strategies. It should be stressed that approximately 80% of obese teenagers will remain so in adulthood [37].

The aim of this study was to evaluate the body mass index (BMI), selected eating behaviour and physical fitness of children aged 10 years attending general education and sports classes in Siedlce. Our working hypothesis was that those schoolchildren which are engaged more in sports will exhibit more appropriate eating behaviour than others. 

## 2. Materials and Methods

### 2.1. Subjects and Procedures

Participants were 10 years old children (mean age 10.8, SD 0.41) at fourth-year grade in primary schools teaching general education classes (GC) or sports classes (SC). The total number of children participating in the study was 285 of which 147 were in GC and 138 were in SC. A correctly completed questionnaire, anthropometric measurements and sport tests results were obtained from 272 children. There were 125 girls and 147 boys, of whom 62 and 77 were in GC and 63 and 70 were in SC, respectively. Physical activity education was 4 and 10 h weekly for GC and SC, respectively [38]. The study was performed during May–June 2018 in 6 primary schools in Siedlce using the Tanita BC-1000 analyser, an in-house questionnaire and the Eurofit test. On the first day, anthropometric measurements and tests of selected eating behaviours were carried out, then the second day was devoted to measure of physical fitness. The study received ethical approval from the Siedlce University Ethic Commission, as well as consent from the child subjects, their parents/legal guardians and the schools’ directors. Exclusion criteria consisted of the following: (1) rejected consent from children or parents, (2) inappropriate age, (3) school absence for 1 or 2 days of the study, (4) poor health/doctor’s sick note and (5) an incorrectly completed questionnaire. None of the subjects were suffering from any acute muscular-skeletal, neurological nor orthopaedic disorders that could affect their ability to carry out the fitness tests. The study was conducted in identical fashion at all of the schools. Segregation into gender and class type was maintained during all measurements. Each child was a priori given an identification number used for later analysis whenever required. Results were gathered and prepared by the study team and then sent to the parents. In 2017, pilot studies were conducted on 10–11-year-old children attending SC and GC (*n* = 50) to check on any assumptions made and the methodology. The methods adopted in the initial study were accepted for the main study. Initial examinations were properly planned and proceeded without issues. There were no changes and modifications during the study.

### 2.2. Questionnaire

A survey questionnaire was devised in-house for the study to evaluate eating behaviour and to gauge the extent of physical activity. The questionnaire was validated before starting a basic study on a smaller group of schoolchildren (*n* = 50). Concept and research plan made in the preliminary study proved succesful and were confirmed in the main study. The method is described further in the text. 

The questionnaire contained 18 choice questions with one answer to choose for each question. Each of the participants was informed about the purpose of the study. A short tutorial was also given to the subjects how to answer each question before filling the questionnaire. The questionnaire was completed in classrooms in the presence of a teacher and a suitable qualified member of the study team to deal with any questions of understanding; the latter being qualified dieticians or graduates from the University of Siedlce. Children had no time limits set. 

Questions dealt with eating behaviour, a self-assessment of nutrition and preferred ways of spending one’s leisure time. Subjects were asked about how often they ate foodstuffs and drinks, such as milk, dairy products, meat, cold meats, fish, white bread, wholemeal bread, groats, rice, fruit, vegetables, drinks, fast-foods and on snacking in-between meals. Five possible answers were given: daily, every 2 days, once a week, once a month or never. In addition, other questions concerned any physical activity performed outside of school-hours, regarding its frequency and passive or active ways of spending leisure time, such as watching television and computer, learning, reading, listening to music, as well as engaging in sports, walking etc. 

A 5-grade scale was used to evaluate behaviour: very good (rank 5), good (rank 4), adequate (rank 3), poor (rank 2) and very poor (rank 1). The scores thus obtained were added up and the marks used to evaluate averages according to each characteristic and class type. High scores thereby reflected beneficial health behaviour. 

The following ranks were assigned to the frequency of consuming those foodstuffs recommended for a healthy diet: daily, every 2 days, once a week, once a month or never. However, for those foodstuffs not recommended in a healthy diet, a reverse grading was adopted. For the types of snacks in-between meals, the highest ranks were given to fruit and vegetables, followed by nuts, while the lowest ones to savouries and confectionery/sweets. For drinks, the highest ranks were given to water, followed by unsweetened fruit-vegetable juices and tea without sugar, whereas the lowest ranks were for fruit juice and soda/carbonated drinks. 

### 2.3. Anthropometric Measurements

Body mass was measured by the Tanita SC-240MA instrument at a precision of 0.1 kg, whereas height (in an erect posture) was measured by the SECA 214 stadiometer at a 1 cm precision. The body mass index (BMI) was calculated with the weight (in kilograms) divided by the squared height (in meters). BMI was compared to percentile charts of Polish children defined in the OLAF and OLA studies [39]. Underweight was defined as a BMI of ≤10 percentile, overweight as ≥85 percentile and obesity by ≥95 percentile. 

### 2.4. Fitness Measurements

This was evaluated by 3 parameters from the European Test of Physical Fitness EUROFIT [31] as follows: (1) torso strength measurement i.e., sit ups for 30 s, (2) running-speeds test/agility i.e., a 10 × 5 m shuttle run, (3) jumping test i.e., a standing long jump. Such tests were supervised by the study team in conjunction with the schools’ physical education teachers. The method of [40,41,42,43] was used to undertake the study and record the results. Two trials were allowed with the best performance of each recorded.

### 2.5. Statistical Analyses

These were performed using Stratasoft Statistica software (version 12) and consisted of descriptive statistics, the T-student, the non-parametric χ^2^-test (chi squared) and Cramer’s V measure of association. The results of fitness tests between SC and GC children were compared using the T-student test. Analysis of nutritional behaviour was calculated using the χ^2^-test (chi squared) and Cramer’s V measure of association test. A α < 0.05 level of statistical significance was adopted throughout, i.e., *p* ≤ 0.05 indicated significant differences, whilst *p* > 0.05 were non-significant differences. The aim of the study was to determine if there were any significant differences in observed eating behaviour, BMIs and fitness measurements between the two study groups (i.e., class types). The strength of association between variables was assessed by Cramer’ V, which is based on the Pearson’s χ^2^ statistic and has an inclusive value of 0 to 1, ranging respectively from no association at 0, and rising to complete association at 1. 

## 3. Results

### 3.1. Group Characteristics

The study participants were 133 SC children (47.3% girls, 52.6% boys) and 139 GC children (44.6% girls and 55.4% boys). The average age of children were 10.8 years old. Participants’ height and weight are listed in Table 1, along with the median, mean, minimum and maximum values, and standard deviations. Large fluctuations in the body weight and height of children in both types of classes were observed, which may indicate a high variability and heterogeneity of the group.

The body weight of SC children ranged from 15.60 kg to 63.20 kg, while the GC ones from 25.70 to 90 kg. The GC children’s mean body mass was 41.60 kg (SD 11.65); this being 42.6 kg and 40.3 kg for boys and girls attending GC classes, respectively. SC subjects’ mean body mass was 38.98 kg (boys 38.9 kg and girls 39 kg; SD 8.54) (Table 1). The mean value of body weight in the classes was at a similar level, while higher values were observed among GC children, and lower values among SC ones, but no significant difference was found (*p* = 0.097).

The mean height for GC children was 147.23 cm (boys 147.68 cm and girls 146.67 cm; SD 7.90), whilst for SC ones, this was 146.03 cm (145.11 for boys and 147.04 cm girls; SD 6.60). GC children were characterised by a higher average height compared to SC ones, yet no statistically significant difference was found.

### 3.2. BMI

The body mass index of the subjects broken down by class and gender is presented in Table 2 along with the median, mean, minimum and maximum values, standard deviations and *p*-value. Large fluctuations were observed in the body mass of children in both classes, especially in GC ones. The BMI of SC children ranged from 13.2 to 25.2 (SD 2.90), while the BMI of GC children was larger, and ranged from 13.30 to 33.90 (SD 3.79). Nevertheless, the mean values of the BMI were similar in both groups (SC 18.21 vs. GC 18.96). There were no statistical differences in BMI of examined children (*p* = 0.061).

It was shown that the BMI of SC and GC girls was classified at a similar level (*p* = 0.437), also SD was at a similar level (SC 2.95 and GC 2.96). BMI of SC girls ranged from 13.20 to 24.80, while GC ones from 14.30 to 25.90. Nevertheless, GC girls had on average higher BMI value than SC girls (GC 18.65 and SC 18.23). The maximum value of the BMI among GC girls was 25.90 and SC ones was 24.80, and the minimum BMI value the opposite (SC 13.20 and GC 14.30).

The BMI of SC boys ranged from 13.80 to 25.20 (DS 2.88), while among GC ones, it ranged from 13.30 to 33.90 (SD 4.35). GC boys were also characterised by a higher mean BMI value (19.21) compared to GC ones (mean BMI 18.19), yet no statistical differences were observed (*p* = 0.100)

It is worth noting that the higher mean value of the BMI among all examined children was observed in GC boys (33.90), while the lowest values were recorded among SC girls (18.23) (Table 2). There were no significant differences in the BMI in relation to the class profile or sex.

Most subjects fell within correct BMI percentile ranges; SC 68.97% and GC 64.88% (Table 3). Healthier body weight was found more often in SC children than in GC ones (SC 68.97% and GC 64.88%). It was the most often recorded in SC boys (72.85%). Underweight was more frequent in SC children (12.85%) than in GC ones (9.88%). Underweight was more often observed among SC girls (14.28%) and among GC boys (11.69%). Overweight and obesity was most often observed in GC children (19.73% and 5.51%, respectively) compared to SC ones (14.37% and 3.8%, respectively). Overweight was on average most often observed among GC girls (22.58%), and then among GC boys (16.88%). Obesity was more frequently reported among GC boys (7.80%) and in SC girls (4.76%). There was no significant difference between girls and boys both at the level of the class profile (*p* = 0.061) and within the sex (SC *p* = 0.935 vs. GC *p* = 0.386).

**Figure 1:**
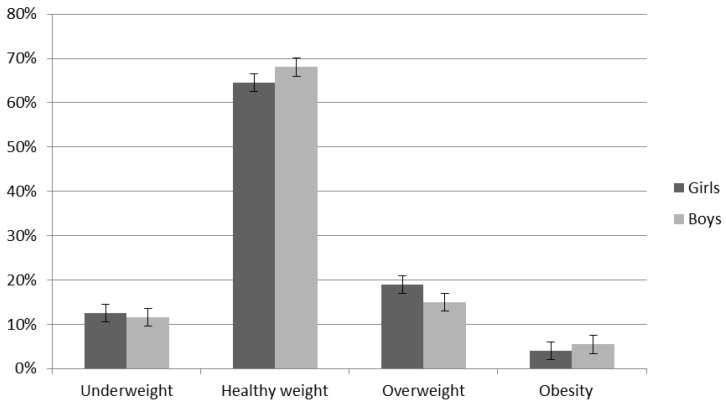
BMI of examined children (all group).

Nearly 70% of the study participants fell within the healthy weight ranges of the BMI percentiles (boys 68.03%, mean 17.75, SD 1.76 vs. girls 64.57%, mean 17.67, SD 1.57) (Figure 1). There was no statistical differences between boys and girls with correct BMI (*p* = 0.754). When analysing the study group by gender, underweight on average was more common among girls (12.59%, mean 14.10, SD 0.54) than boys (11.56%, mean 14.42, SD 0.52), which, in the average classification, was 11.40% and not significant (*p* = 0.11). Overweight was observed on average among nearly 17% of all participants of the study; interestingly, overweight was more common among girls (18.90%, mean 22.31, SD 0.74) than among boys (14.97%, mean 22.79, SD 1.05), but it was not significant (*p* = 0.080) as well, while obesity was the opposite (boys 5.44%, mean 28.87, SD 4.09 vs. girls 3.94, mean 24.54, SD 0.83), with an average BMI of 4.78% for the whole group. It was significantly more for boys with obesity than girls (*p* = 0.04).

### 3.3. Eating Behaviour

Eating behaviour was identified (Table 4). High numerical values reflected favourable eating behaviour. SC children had insignificantly higher mean values for the ‘very good’ (36.78%) and ‘good’ (20.57%) gradings compared to GC, which were 33.65% and 19.68%, respectively. SC children more frequently consumed poultry (*p* = 0.639), vegetables (*p* = 0.044), fish (*p* = 0.013), wholemeal bread (*p* = 0.001), groats, wholegrain pasta etc. (*p* = 0.015) milk and fermented dairy drinks (*p* = 0.006) as compared to GC. 

The number of daily meals was similar in SC and GC groups (*p* = 0.616). Snacking in-between meals mostly consisted of fruit (GC 35.97% and SC 41.35%). GC children more often snacked on confectionery (33.81%) and snacks (26.62%), when compared to SC children at respectively 24.81% and 18.80% (*p* = 0.003). 

An average of 62.31% subjects declared eating dairy products daily (GC children at 53.96% and SC at 70.68%; *p* = 0.006).

Fruit and vegetables were eaten once a day on average. SC children ate significantly more fruit and vegetables than GC. Daily vegetable consumption for GC and SC were respectively 42.45% and 49.62% (*p* = 0.044). Fruit decidedly more often featured in diets than vegetables, nevertheless, the frequencies varied (*p* = 0.163). 

The frequency of white bread consumption declared by children was high, at an average daily rate of 60.57% (GC at 64.75% and SC at 56.39%; *p* = 0.632), whereas wholemeal bread 22.51% (GC at 18.71%, SC at 26.32%; *p* = 0.001). Despite the low levels of wholemeal bread consumption, rates were significantly higher for SC children than GC ones. 

Coarse-grained groats, porridge oats and wholegrain pasta were rarely eaten, with only 9.96% being consumed overall daily (GC at 8.63% and SC at 11.28%; *p* = 0.015). Over two-thirds of subjects, however, ate white rice, fine-grained groats and pasta (GC at 61.87% and SC at 60.15%; *p* = 0.952). 

The frequency of poultry meat consumption by children was low, but on average, SC children more often declared consumption of it than GC ones. Almost 45% subjects ate poultry every day (GC 41.73% and SC 47.37%; *p* = 0.639).

Children clearly ate more frequently poultry than red meat, with the latter being most often consumed every other day at a mean rate of 32.3% (GC 34.53 and SC 30.08%; *p* = 0.000); the once weekly rate being 27% (GC 20.14% and SC 33.83%). 

Frequency of fish consumption was low, where the once a week rates were 64.77% (GC at 61.87% and SC 67.67%; *p* = 0.013) and once a month at 20.65%, whilst 14.58% never ate fish. Fish consumption was at low levels irrespective of class type, nevertheless SC children ate significantly more fish than GC.

Water was the most commonly drank beverage for both groups (rank 5) (GC 42.44% and SC 49.62%; *p* = 0.278). Non-sugared tea was drunk by 22.56% SC children and 15.83% GC. 

The highest rates of fast food consumption were at once a month 66% (GC at 63.31% and SC at 67.67%; *p* = 0.695), whereas those never eating fast food were above 20% for both GC and SC.

On average, SC children demonstrated more favourable beverage consumption behaviour than GC ones, although this was statistically insignificant. 

### 3.4. Extra-Curricular Sports Activity

**Figure 2:**
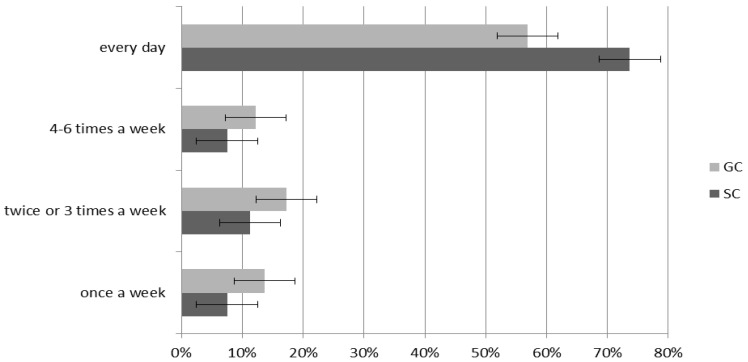
The frequency of sport activity after the school, times a week.

Children most commonly declared that they prefer to spend their leisure time engaging in sport: 82.8% on average (SC 86.47% and GC 79.14%), whereas 17.2% preferred passive activities like watching TV (SC 13.53% and GC 20.86%; *p* = 0.011). Our study showed that all children were physically active and preferred sports participation out of school, nonetheless the rates varied (Figure 2). SC children more often preferred to participate in sports, with an overall 65.26% declaring that they do so every day (GC 56.83% and SC 73.68%). GC children were more likely declared sports activity four to six times a week (GC 12.23% and SC 7.52%) and twice or three times a week (GC 17.27% and SC 11.28%). On average, 10.59% of children declared that they were only sport active one day a week (GC 13.67% and SC 7.52%). Statistical analysis showed significant differences (χ^2^ = 15.09, df = 4, *p* = 0.05, VC = 0.17).

A decisive majority (overall 90.84%; SC 92.48% and GC 89.21%), were well aware of the beneficial effects that healthy nutrition on sport achievement (χ^2^ = 0.872, df = 1, *p* = 0.350, VC = 0.05). Most children declared eating healthily, with GC at 64.03% and SC at 58.65%, whilst, in contrast, 38.66% considered their nutrition to be unhealthy (GC 35.97% and SC 41.45%). The differences were significant (χ^2^ = 0.831, df = 1, *p* = 0.03, VC = 0.05). 

### 3.5. Fitness Measurements

In all measures of fitness tests, i.e., shuttle run, long jump and the number of neighbours from lying down children from the sports class achieved statistically significantly better results. Table 5 shows the differences between SC and GC. The SC children achieved better mean results than GC in all tests. Large fluctuations were observed in all tests where SD is very large, especially in the standing long jump attempt. 

In the standing long jump test for example, SC children scored 141.03 cm (range 195–100 cm), whereas GC scored 135.02 cm (range 180–98.00 cm). SC children achieved on average better results in the long jump test compared to GC ones (*p* = 0.022). In this test large fluctuations in SD (SC 19.85 vs. GC 22.93) were recorded, which proves the large group volatility.

In the SC group, the number of sit-ups in 30 seconds ranged from 15 to 32 (SD 2.72) and from to 2 to 27 (SD 4) in GC. On average, SC children achieved better results in the torso strengths test (SC 22.58 and GC 20.05) (p = 0.000). The lowest recorded result in GC was only 2, in comparison to with the number SC 15.

In the shuttle run test, SC children obtained an average better time compared to GC ones (SC 21.62 sec. and GC 23.02 sec.). SC children achieved an average time ranging from 17.00 to 29.03 (SD 2.24), while GC ones from 17.89 to 32.09 (SD 2.70) (*p* = 0.000).

In addition, test results were compared between girls from the SC and GC classes and boys from both classes (Table 6). In the case of girls, statistical differences were in the results of the standing long jump (*p* = 0.005) and sit-ups (*p* = 0.000). Boys results were on the shuttle run (*p* = 0.003) and also sit-ups (*p* = 0.008). Both girls and boys from SC performed better than their GC peers.

## 4. Discussions

Both an inadequate diet and lack of physical activity among school children, may increase the risk of obesity and associated chronic disease. Obesity is one of the main risk-factors responsible for multi-aetiological pathologies [44,45]. Our study showed that children, on average, had correct body mass (SC 68.97 vs. GC 64.88), however, overweight and obesity were more frequent among GC children (ca. ¼ of children) than SC ones (ca. 1/5). Underweight was less often reported and concerned more often SC children than GC ones. Many studies showed that low physical activity is often positively associated with overweight [46,47]. A study by [48] demonstrated that BMI is significantly associated with physical activity during puberty (13–18 years) in boys and in pre-pubertal girls aged 7–12 years [48]. A study by [49], however, found that there was no significant relationship between the type of class (i.e., general education vs. sports focused). in the incidence of overweight and obesity based on BMI [49], which is consistent with our results (Table 2), where the most children fell within healthy weight BMI percentile ranges (Table 3), but generally much weaker physical condition was observed in GC. 

Children from medium-sized town in this study attending profiled sports classes promise good public health indicators in adult life compared to children attending general classes. It is worth emphasising that Siedlce is a small city where sporting events and additional sports activities are limited, but if someone is motivated, they can lead an active and healthy lifestyle. If children with GC do not play sports, they will have health problems in the future. At this time, there are no differences in body weight and BMI, but we can see weaker physical fitness in GC. Unfortunately, we do not have studies presenting the occurrence of diet-related diseases among adults living in Siedlce, but we can expect an upward trend.

Our results showed that SC children achieved higher average ranks in the ‘very-good’ and ‘good’ gradings regarding eating behaviour when compared to GC children. Advantageous choices in nutrition most commonly made by children SC and GC were: eating at least four daily meals, preferring water and unsweetened beverages, eating fruit at least once a day, consuming milk and dairy products and, only very occasionally, eating fast food (once a month). Such behaviours are linked inter alia to recommendations on keeping adequately hydrated (with focus on intakes of water) and drinking fruit, vegetable and fruit-vegetable drinks [50,51], as well as eating fruit per se. Nonetheless, a small group of children did not keep to the recommended dietary intakes of daily wholegrain products and vegetables. An even smaller proportion of subjects ate coarse-grained groats wholegrain pasta and porridge oats. The dietary abnormalities and qualitative errors in nutrition found in this study were in fact similar to other studies [50,52,53,54]. Many studies indicate a positive association between the extent that the principles of a healthy diet and physical activity [55,56]. Furthermore, a study by Lipsky et al. [57] and Noll et al. [58] showed that a better-quality diet was associated with higher physical activity [57,58]. A study by [59] and [60] found the more frequent physical activity the higher meat, fish, wholemeal bread, vegetables and fruit intake [59,60]. 

For children and young people practicing sports. The diet should be based on dietary guidelines. Those who exercise regularly, have increased nutritional needs, especially with regard to energy, protein and fluids. The nutrient requirements depend on gender stage of growth level of training and type of sport [61]. Using various dietary models, MD has been shown to be one of the best-studied diets [62,63,64]. Children spending more time on sports more often consumed whole grains, fish, poultry, vegetables, fruit and water

Reinforcing efforts to promote healthy diets and physical activity in children and adolescents are necessary for improving the health of future adult populations. Such actions should be undertaken by both educational and health care institutions. A study by [65] found that adolescents who exercised more frequently had a superior health status. Such behaviour was observed in our study (Table 5). Just 4 h of PE lessons at the GC are often insufficient to maintain adequate physical fitness. For example a study by [22] emphasises the important contribution that sport leads to numerous health benefits, such as improved aerobic fitness, muscle strength and endurance, increased bone mineral density and a more advantageous profile of cardiovascular risk factors.

Analysing the results of fitness testing much better results were observed among the SC children than GC ones. A similar observation was in the research by [66] involving a group of 293 children. It shows that compliance with the guidelines for physical activity results in better sit and reach test and right handgrip strength test. In the above study, higher BMI was associated with poorer performance in most of the assessed fitness tests. Moreover, it was found, that overweight children, compared to peers with normal body weight, were characterised by weaker results in tests requiring propulsion or weight-lifting: horizontal jump, 10 × 5 m agility run and endurance shuttle run [66]. The above confirmation can also be found in the study conducted by [8] and [67], in which the participants with the highest performance in tests of physical condition (especially in terms of cardio-respiratory endurance) were people who were characterised by compliance with MD recommendations. 

An extensive study on over 47,000 children and adolescents aged 10–18 years from 18 European countries indicated that two-thirds were insufficiently physically active, whilst only one-third could be considered to be satisfactorily active in this respect [68]. Our study demonstrated that subjects had high levels of physical activity, which was confirmed by the survey questionnaire, where more than 80% of children preferred active forms of spending their leisure time, and more than two-thirds engaged in extra-curricular sports every day. It should be stressed that SC children were more physically fit than GC ones, which significantly translated into sports achievements. The study by [69] and [70] reported significantly better outcomes for physical fitness tests in children who were constantly taking part in sports club activities when compared to their peers who were not attending. The study by [71] studied changes in physical fitness and body composition throughout two seasons in 11-year-old footballers, and these data were compared to an age-matched control group. The results showed that the former achieved superior parameters of physical fitness and body composition compared to controls particularly in terms of speed (i.e., 10 m and 30 m sprint) agility (i.e., running) endurance (swinging run), which we can also find in our study. We can expect that physical education will in the future influence better results of sport tests and anthropometric parameters. This thesis was confirmed in a study by [72], which assessed the impact of long-term sports training on physical fitness and body composition in SC children, which was compared to their GC counterparts [71]. It was found that SC children showed greater improvements in physical fitness (including a 20 m sprint, long jump test, agility test) after engaging in regular physical education and additional sports training, compared to the GC children.

The analysis showed that the more hours the surveyed students devoted themselves to sport during the day, the more often they ate poultry meat, fish, vegetables and fruits, whole-grain bread and coarse cereals, whole-grain pasta, etc. The increase in the number of hours of physical exercise correlated with sports achievements. The children’s nutritional behaviours indicate a constant need for observation and education of this group of people, in order to eliminate the bad eating habits repeated in many studies.

### Strengths and Limitations

The questionnaire was limited to the question concerning the frequency of eating of various food products, without taking into account the amount of foods consumed, and has not been subject to criterion-based validity testing. Secondly, it is well known this socioeconomic status can affect the acquisition of good healthy habits, but this was not taken under consideration in this study. The study was limited only to the sample of children attending grade 4 primary schools in Siedlce. Moreover, children sport participation out of school was evaluated only by asking about preferences in this regard. However, the strength of the study is the selection of two groups: SC and GC consisting of girls and boys aged 10 attending grade 4. The number of students in both groups was similar and the parameters analysed were measured using the same tests by the same research team, which, in turn, reduces the risk of irregularities.

## 5. Conclusions

The type of class attended by the children impact on eating behaviour, BMI and physical fitness level. More correct nutritional behaviour was observed among SC children regarding the consumption of vegetables, fish, white meat, wholemeal bread, milk and dairy products. In contrast, GC children much more often declared eating sweets, snacks and drinking carbonated drinks compared to SC ones. Most children fell within the healthy weight BMI percentile charts. However, overweight and obesity was more frequent among GC children. SC children were more fit, had healthier BMIs, and more often exhibited appropriate eating behaviour than GC ones. Our results confirmed that increasing the number of children’s sports activity level has the desired health effect. It is therefore necessary to develop suitable educational programmes promoting the principles of healthy nutrition and sport activity among schoolchildren. 

## Figures and Tables

**Table 1:** Group characteristics.

Variable	Class	Average	Median	Min.	Max.	SD *	*p*
Body Weight	SC	38.98	37.60	15.60	63.20	8.54	*p* = 0.097
	GC	41.60	39.40	25.70	90.00	11.65	
Height	SC	146.03	145.00	128.00	168.00	6.60	*p* = 0.182
	GC	147.23	146.00	129.00	170.00	7.90	

* SD—standard deviation.

**Table 2:** Characteristics of the group body mass index (BMI).

Variable	Class	Average	Median	Min.	Max.	SD *	*p*
BMI	SC	18.21	17.80	13.20	25.20	2.90	*p* = 0.061
	GC	18.96	18.20	13.30	33.90	3.79	
BMI Girls	SC	18.23	18.00	13.20	24.80	2.95	*p* = 0.437
	GC	18.65	18.15	14.30	25.90	2.96	
BMI Boys	SC	18.19	17.65	13.80	25.20	2.88	*p* = 0.100
	GC	19.21	18.40	13.30	33.90	4.35	

* SD—standard deviation.

**Table 3:** Body mass of children from general and sports classes according to BMI percentile grids [%].

Variable	Underweight		Healthy Weight		Overweight		Obesity	
**SC**								
	%	*p*	%	*p*	%	*p*		*p*
Total Population	12.85	-	68.97	-	14.37	-	3.80	-
Girls	14.28	0.019	65.09	0.782	15.87	0.161	4.76	0.095
Boys	11.43		72.85		12.87		2.85	
**GC**								
Total Population	9.88	-	64.88	-	19.73	-	5.51	-
Girls	8.07	0.589	66.13	0.491	22.58	0.297	3.22	0.132
Boys	11.69		63.63		16.88		7.80	

**Table 4:** Nutritional behaviour of sports classes (SC) and general education classes (GC) children [%].

Lp.	Nutritional Behaviour *n* = 272	SC					GC					χ^2^
		Ranks in a 5-Point Scale (% of Particular Ranks in the Group) *										
		1	2	3	4	5	1	2	3	4	5	
1.	Number of meals eaten during the day	0.00	0.00	7.52	54.89	37.59	0.00	0.00	10.79	54.70	34.50	χ^2^ = 0.96, df = 2, *p* = 0.616 VC = 0.05
2.	Type of food eaten between main meals	24.81	18.80	0.00	41.35	15.04	33.81	26.62	0.00	35.97	3.60	χ^2^ = 13.88, df = 3, *p* = 0.003 VC = 0.22
3.	Eating of milk and dairy products	0.00	0.00	6.77	22.56	70.68	0.00	6.47	14.39	25.18	53.96	χ^2^ = 15.95, df = 5, *p* = 0.006 VC = 0.24
4.	Eating of vegetables	12.78	0.00	4.51	33.08	49.62	14.39	0.00	8.63	34.53	42.45	χ^2^ = 2.67, df = 3, *p* = 0.044 VC = 0.09
5.	Eating of fruit	0.00	0.00	3.01	22.56	74.44	0.00	0.00	9.35	17.99	72.66	χ^2^ = 5.10, df = 3, *p* = 0.163 VC = 0.13
6.	Eating of white bread	0.00	11.28	12.78	19.55	56.39	0.00	7.19	12.23	15.83	64.75	χ^2^ = 2.56, df = 4, *p* = 0.632 VC = 0.09
7.	Eating of wholemeal bread	22.56	6.02	33.83	11.28	26.32	25.90	21.58	28.78	5.04	18.71	χ^2^ = 17.69, df = 4, *p* = 0.001 VC = 0.25
8.	Eating of thick groats, wholegrain pasta etc.	11.28	14.29	45.11	18.05	11.28	18.00	6.47	48.20	18.71	8.63	χ^2^ = 6.74, df = 4, *p* = 0.015 VC = 0.15
9.	Eating of small groats, ordinary pasta etc.	0.00	0.00	17.30	22.56	60.15	0.00	0.00	19.42	18.71	61.87	χ^2^ = 0.69, df = 4, *p* = 0.952 VC = 0.05
10.	Eating of poultry meat	8.27	11.28	21.05	12.03	47.37	10.79	7.91	25.18	14.39	41.73	χ^2^ = 2.52, df = 4, *p* = 0.639 VC= 0.09
11.	Eating of red meat	11.28	8.27	33.83	30.08	16.54	12.59	6.47	20.14	34.53	25.90	χ^2^ = 8.41, df = 4, *p* = 0.000 VC = 0.17
12.	Eating of fish	9.02	23.31	67.67	0.00	0.00	20.14	17.99	61.87	0.00	0.00	χ^2^ = 10.76, df = 4, *p* = 0.013 VC = 0.160
13.	Beverages	13.53	14.29	22.56	0.00	49.62	21.58	20.14	15.83	0.00	42.44	χ^2^ = 6.30, df = 5, *p* = 0.278 VC = 0.15
14.	Eating of fast food	22.56	67.67	9.77	0.00	0.00	20.86	63.31	15.83	0.00	0.00	χ^2^ = 2.22, df = 4, *p* = 0.695 VC = 0.09
Mean		9.72	12.51	20.40	20.57	36.78	12.69	13.15	20.76	19.68	33.65	

* ranks: 5—very good, 4—good, 3—medium correct, 2—small, 1—very low.

**Table 5:** Eurofit performance test results.

Variable	Class	Average	Median	Min.	Max.	SD *	*p*
10 × 5 m Shuttle Run (s)	SC	21.62	21.70	17.00	29.03	2.24	*p* = 0.000
	GC	23.02	22.66	17.89	32.09	2.70	
Standing Long Jump (cm)	SC	141.03	140.00	100.00	195.00	19.85	*p* = 0.022
	GC	135.02	135.00	98.00	180.00	22.93	
sit-ups in 30 (n/30 s)	SC	22.58	22.00	15.00	32.00	2.72	*p* = 0.000
	GC	20.05	20.00	2.00	27.00	4.00	

* SD—standard deviation.

**Table 6:** Eurofit performance test results with division sex.

Variable	Sex Class	Average	Median	Min.	Max.	SD *	*p*
10 × 5 m Shuttle Run (s).	Girls SC	23.40	23.39	20.18	29.03	1.60	*p* = 0.159
	Girls GC	23.98	23.81	18.95	32.09	2.84	
	Boys SC	21.12	21.30	16.35	26.60	2.08	*p* = 0.003
	Boys GC	22.21	21.850	17.89	32.00	2.30	
Standing Long Jump (cm).	Girls SC	139.53	140.00	100.00	195.00	19.45	*p* = 0.005
	Girls GC	130.17	130.00	88.00	170.00	17.82	
	Boys SC	142.37	140.00	100.00	190.00	20.24	*p* = 0.321
	Boys GC	138.61	140.00	0.98	180.00	25.07	
Sit-Ups in 30 s (n/30 s).	Girls SC	22.30	22.00	15.00	29.00	2.42	*p* = 0.000
	Girls GC	18.43	18.00	6.00	25.00	3.51	
	Boys SC	22.84	22.500	18.00	32.00	2.95	*p* = 0.008
	Boys GC	21.31	22.00	2.00	27.00	3.91	

* SD—standard deviation.
